# Gene polymorphisms of insulin secretion signaling pathway associated with clopidogrel resistance in Han Chinese population

**DOI:** 10.1002/jcla.23970

**Published:** 2021-10-05

**Authors:** Jinyan Zhong, Qinglin Yu, Nan Zheng, Jia Su, Xiaowei Zheng, Liangrong Zheng, Xiaomin Chen

**Affiliations:** ^1^ Zhejiang University School of Medicine Hangzhou China; ^2^ Department of Cardiology Ningbo Second Hospital Ningbo China; ^3^ Department of Traditional Chinese Internal Medicine Ningbo First Hospital Ningbo China; ^4^ Department of Cardiology Ningbo First Hospital Ningbo China; ^5^ Department of Geriatrics Ningbo First Hospital Ningbo China; ^6^ Department of Cardiovascular Sciences First Affiliated Hospital Zhejiang University School of Medicine Hangzhou China

**Keywords:** clopidogrel resistance, insulin secretion, signaling pathway genes, SNP

## Abstract

**Background:**

Due to the loss of responsiveness to insulin, diabetes mellitus (DM) patients develop increased platelet reactivity and reduced response to antiplatelet agents. Nevertheless, the relationship between the single‐nucleotide polymorphisms (SNP) of the signal pathway gene of insulin secretion and the effect of clopidogrel is elusive.

**Methods:**

Blood samples were collected from patients administered with dual‐antiplatelet therapy (clopidogrel, 75 mg, once daily and aspirin, 100 mg, once daily) after 5 days and completed test within 4 h. The VerifyNow P2Y12 assay was used to measure the platelet functions, and the results were expressed as a P2Y12 reaction unit (PRU). Notably, the selected SNPs were analyzed to demonstrate the functionality of genetic variants.

**Results:**

Analysis of the study population showed that old age, lower plasma albumin (ALB) level, higher creatinine (CREA) level, higher uric acid (UA) level, lower platelet (PLT) count, and lower plateletcrit (PCT) potentially increased the risk of clopidogrel resistance. In a single‐nucleotide polymorphism rs6056209 of the PCLB1 gene, the AG genotype was a risk factor for clopidogrel resistance (*p* < 0.05, OR = 1.574). Similarly, the CC and AG genotype in GNAS rs7121 and CCKAR rs1800857 were protective factors (*p* < 0.05, OR = 0.094; *p* <0.05, OR = 0.491). TT was a protective factor in rs10814274 of the CREB3 gene (*p* < 0.05, OR = 0.444). In the RAPGEF4 gene polymorphism rs17746510, TG was the protective genotype, and the TT genotype was a risk factor for clopidogrel resistance. GCG rs5645 was confirmed; there was a relationship between genotypes containing A or G and clopidogrel resistance.

**Conclusion:**

Single‐nucleotide polymorphisms of insulin secretion signaling pathway genes trigger clopidogrel resistance.

## Graphical abstract

The association between the clopidogrel resistance and gene polymorphisms of insulin secretion signaling pathway, including FXYD2, GCK, PCLO, ATF6B, CACNA1S, PLCB1, GNAS, KCNMA1, CCKAR, CREB5, GCK, YKT6, YKT6, GCK, PCLO, STX1A, GNA11, GCG, RAPGEF4, and CREB3.

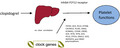

## 1 INTRODUCTION

Dual‐antiplatelet therapy with clopidogrel and aspirin is widely used in the prevention of blood clot formation in ST‐elevation myocardial infarction (STEMI) and non–ST‐elevation myocardial infarction (NSTEMI) patients.^1^ Clopidogrel is a prodrug that irreversibly binds to the P2Y12 receptor on the platelet surface. Platelet activity inhibition in 5%–44% of patients is significantly low, a phenomenon described as clopidogrel resistance.^2^, ^3^ Interindividual variability in drug response is one of the challenges in antiplatelet treatment. The disposition, metabolism, transporters, or targets of a drug affected by polymorphisms are implicated in an individual antithrombotic drug modification specifically, clopidogrel. Studies on the mechanisms causing interindividual variability in drug response are limited^4^; both genetic and non‐genetic factors must be considered.^5^ Single‐nucleotide polymorphisms are the most prevalent genetic variation in the human genome. More than 9 million SNPs have been reported in public databases.^6^ SNPs including promoters, exons, introns, and 5′‐ and 3′UTRs are located in different regions of genes. Different regions of SNPs potentially influence gene expression by changing promoter activity, binding transcription factors, DNA CpG site methylation, histone modifications, and suppressing gene transcription and translation.^7^ SNPs in the 5′‐UTR affect translation, while SNPs in the 3′‐UTR influence microRNA (miRNA) binding. Researchers revealed that several SNPs in the beta cell genes regulate insulin secretion.^8^

Interindividual response heterogeneity is linked to several non‐genetic factors including age, renal and liver function, diabetes mellitus, and smoking by up‐regulation of platelet‐signaling pathways. Studies indicate that due to the loss of responsiveness to insulin, DM patients develop increased platelet reactivity and reduced response to antiplatelet agents.^9^ Patients diagnosed with DM require more effective antiplatelet drugs than patients without DM despite under treatment with clopidogrel and aspirin (ASA).^10^ Insulin receptor substrate‐1 (IRS‐1) is a central role in the insulin signal transduction pathway and affects Ca^2+^ regulating mechanisms in DM patients.^11^

Notably, the primary cause of diabetes is insufficient insulin secretion, whether absolute or relative. Insulin secretion is closely related to the signaling pathway. All genes including (*FXYD2*, *GCK*, *PCLO*, *ATF6B*, *CACNA1S*, *PLCB1*, *GNAS*, *KCNMA1*, *CCKAR*, *CREB5*, *GCK*, *YKT6*, *YKT6*, *GCK*, *PCLO*, *STX1A*, *GNA11*, *GCG*, *RAPGEF4*, and *CREB3*) are in the signaling pathway. Previous research confirmed the relationship between GNAS and obesity.^12^ So far, studies on the relationship between clopidogrel resistance and the polymorphic variants of the insulin secretion gene have not reached maturity. Therefore, this work aims to investigate the relationship between genetic variants of the insulin secretion gene and clopidogrel resistance.

## 2 METHODS

### 2.1 Study population

In total, this study consecutively enrolled 210 patients with acute coronary syndromes from Ningbo First Hospital between 2015 and 2018. These patients were of Han ethnicity and lived in Ningbo City, Zhejiang Province for more than 10 years. Inclusion criteria include the following: over 18 years old; received a loading dose of clopidogrel and aspirin before PCI, and were daily administered with dual‐antiplatelet therapy after stent placement. Meanwhile, patients were excluded if they had known liver or kidney failure; had been receiving anticoagulation therapy with warfarin and other anticoagulant drugs; had a history of severe bleeding or abnormal platelets (<150,000 μl^−1^ or >500,000 μl^−1^). This study conformed to the ethical guidelines of the Helsinki declaration.^13^ The ethics approvals were provided by the Ningbo First Hospital ethics committee, and all patients provided their informed written consent.

### 2.2 Platelet function measurements

Based on previous related studies, 3 ml venous blood was drawn from patients administered with dual‐antiplatelet therapy (clopidogrel, 75 mg, once daily and aspirin, 100 mg, once daily) after 5 days, and completed the test within 4 h.^14^ The VerifyNow P2Y12 assay was applied to measure the platelet functions, and the results were expressed as a P2Y12 reaction unit. PRU ≥240 was considered clopidogrel resistance.^14^

### 2.3 DNA extraction and genotype testing

Human genomic DNA was extracted from 3 ml of peripheral blood using QIAamp‐DNA Serology Kit (Qiagen).^15^ (a) The sample was stored in the refrigerator for several days. Exactly 3 ml blood sample was drawn and placed in a new vacuum collection tube. (b) Then, red blood cell lysate was added, mixed thoroughly, centrifuged at 3000 *g* for 2 min, and then, the supernatant was discarded. (c) Step 2 was repeated twice until the content turned into a white precipitate. (d) After shaking the test tube, cells were observed in the suspension. Then, the white blood cell lysate was added, and the shaker was shaken for 30 s, then the cells remained suspended on it. (e) The protein precipitation solution was added, and a red flocculent precipitate was observed after shaking. The mixture was centrifuged at 12,000 *g* at 4°C for 10 min. (f) The supernatant was added to the EP test tube; then an equal volume of isopropanol was added, inverted, and mixed several times, until a flocculent precipitate was observed. Centrifugation was performed at 12,000 *g* at 4°C for 10 min. The supernatant was discarded. (g) Exactly 500 μl 70% ethanol was added to the EP tube, then subjected to washing. The procedure was reversed several times. After floating the precipitate, centrifugation was conducted at 12,000 *g* at 4°C for 10 min. The supernatant was discarded and the procedure was repeated. (h) The mixture was dried at a constant oven temperature of 40°C for about half an hour. Thereafter, DNA dissolving solution was added to the EP tube, mixed, and dissolved thoroughly. Primers were designed by PyroMark Assay Design software. The product was amplified via polymerase chain reaction (PCR). Afterward, the amplified product was purified and sequenced.

### 2.4 Statistical analysis

Statistical analysis was performed using SPSS version 26.0 (SPSS, Somers). A chi‐square test was used to establish whether genetic polymorphisms were at Hardy‐Weinberg equilibrium. Continuous variables conformed to a normal distribution and were described as mean ± standard deviation. Non‐normally distributed variables were presented as the interquartile range (IQR). As appropriate, Pearson's chi‐square test was used for categorical variables. The Wilcoxon rank‐sum test was adopted to evaluate nonparametric continuous variance. Results with *p*‐value < 0.05 were considered statistically significant.

## 3 RESULTS

### 3.1 Analysis of clinical characteristics or baseline data

The results of clinical characteristics and baseline data of clopidogrel‐resistant and non‐resistant groups are shown in Table 1. Platelet function analysis was performed in 210 patients. A total of 96 patients with PRU ≥240 were defined as clopidogrel resistance. On the other hand, 114 patients with PRU <240 belonged to the non‐resistant group. A tendency toward clopidogrel resistance was noted for the following: Age (case and control group: 66 [54–73.75] versus 60 [51–67.25], *p* = 0.001); ALB levels (case and control group: 38.2 [36.05–40.1] versus 40.6 [37.7–42.3], *p* < 0.001); CREA levels (case and control group: 76 [64.5–85.6] versus 67 [63.2–73.8], *p* = 0.001); UA levels (case and control group: 333 [279–409] versus 278 [152–333], *p* < 0.001); PLT levels (case and control group: 188 [137–219] versus 204 [161.75–248], *p* = 0.001); and PCT levels (case and control: 0.15 [0.13–0.1775] versus 0.16 [0.14–0.21], *p* <0.001). The factors including (old age, lower plasma ALB level, higher CREA level, higher UA level, lower PLT count, and lower PCT) might affect the risk of clopidogrel resistance. A total of 24 preselected SNPs were genotyped and most of them did not depart from the Hardy–Weinberg equilibrium (HWE) except five SNPs, which were not in HWE; they include *FXYD2 rs12286470*, *GCK rs1799884*, *PCLO rs2715148*, *ATF6B rs8283 and CACNA1S rs2365293*.

**TABLE 1:** Statistics of clinical characteristics of the study population.

Characteristics	non‐CR (*n* = 114)	CR (*n* = 96)	*Z*/*X* ^2^	*p* value
Age, year	60 (51–67.25)	66 (54–73.75)	12.018	0.001
BMI, kg/m^2^	23.66 (20.7–27.1)	24.0 9(22.8825–25.14)	0.270	0.603
TC, mg/dl	4.395 (3.795–5.68)	4.17 (3.76–4.97)	0.787	0.375
TG, mg/dl	1.35 (1.0725–2.22)	1.405 (0.93–1.6375)	2.248	0.134
HDL, mg/dl	0.93 (0.7975–1.1)	0.99 (0.76–1.19)	1.236	0.266
LDL, mg/dl	2.555 (2.03–3.5)	2.36 (1.95–2.99)	1.238	0.266
GLU, mmol/L	5.14 (4.6825–5.79)	5.375 (4.6–7.16)	1.794	0.180
ALT, μmol/L	26 (16–47.25)	23.5 (15–42)	0.960	0.327
AST, μmol/L	25 (18–135.25)	26 (18–77)	0.170	0.680
TBIL, μmol/L	11.25 (9.3–16.1)	11.15 (7.075–25.425)	0.007	0.935
ALB, g/L	40.6 (37.7–42.3)	38.2 (36.05–40.1)	12.997	<0.001
BUN, mmol/L	5.2 (4.01–6.74)	5.265 (4.85–6.655)	1.360	0.244
CREA, mmol/L	67 (63.2–73.8)	76 (64.5–85.6)	10.519	0.001
UA, μmol/L	278 (152–333)	333 (279–409)	18.904	<0.001
hsCRP, mg/L	1.84 (0.9925–8.47)	3.32 (0.5975–10.295)	0.024	0.877
PLT*10^9^/L	204 (161.75–248)	188 (137–219)	11.243	0.001
MPV, fL	8.1 (7.3–9.525)	8.1 (7.4–9.175)	0.101	0.750
PCT, %	0.16 (0.14–0.21)	0.15 (0.13–0.1775)	13.964	<0.001
PDW, %	16.2 (15.6–16.6)	16.3 (15.95–16.5)	2.700	0.100
HbA1c, %	5.95 (5.65–6.3)	5.7 (5.5–6.675)	0.085	0.771
Gender (male) *n* (%)	2 7(23.7)	69 (71.9)	1.074	0.300
Hypertension, *n* (%)	27 (23.7)	69 (71.9)	1.463	0.226
Diabetes mellitus, *n* (%)	78 (68.4)	18 (18.8)	0.754	0.385
Hyperlipidemia, *n* (%)	38 (33.3)	32 (33.3)	0.000	1.000
Smoke, *n* (%)	48 (42.1)	34 (35.4)	0.980	0.322
Alcohol abuse, *n* (%)	21 (18.4)	15 (15.6)	0.287	0.592

Note

The significant values are marked in bold (*p* ≤ 0.05).

Abbreviations: ALB, albumin; ALT, Alanine aminotransferase; AST, Aspartate aminotransferase; BUN, blood urea nitrogen; CREA, creatinine; GLU, Glucose; HbA1C, glycated hemoglobin; HDL‐C, high‐density lipoprotein cholesterol; hs‐CRP, high sensitive C reactive protein; LDL‐C, low‐density lipoprotein cholesterol; MPV, Mean platelet volume; PCT, Platelet hematocrit; PDW, Platelet distribution width; PLT, platelet; TBIL, total bilirubin; TC, Total cholesterol; TG, Triglyceride; UA, Uric acid.

**FIGURE 1:**
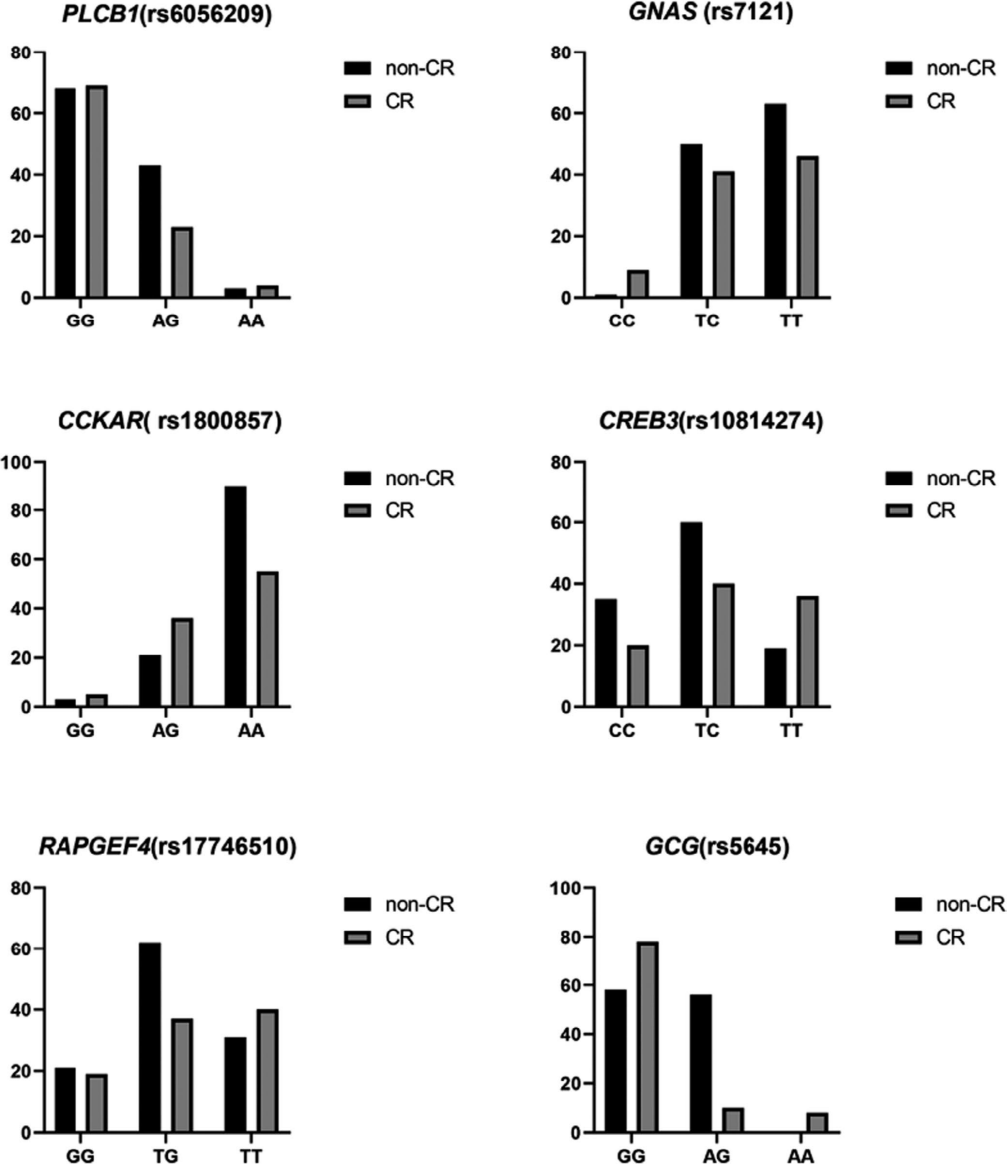
The frequency of alleles and genotypes of PCLB1 rs6056209. GNAS rs7121. CCKAR rs1800857. CREB3 rs10814274. RAPGEF4 rs17746510 and GCG rs5645. *p < 0.05

In multiple single‐nucleotide polymorphisms of multiple genes in the insulin‐related secretion pathway (Table 2), a few genotypes were related to clopidogrel resistance. In the single‐nucleotide polymorphism *rs6056209* of the *PCLB1* gene, the AG genotype was statistically significant (*p* < 0.05) and a risk factor for clopidogrel resistance (OR = 1.574). Similarly, in *GNAS rs7121*, the CC genotype was a protective factor (*p* < 0.05, OR = 0.094). In *rs1800857* of the *CCKAR* gene, AG was also a protective factor (*p* < 0.05, OR = 0.491). In rs10814274 of *CREB3* gene, TT was a protective factor (*p* < 0.05, OR = 0.444). In the *RAPGEF4* gene polymorphism *rs17746510*, TT was the protective genotype (*p* < 0.05, OR = 0.653), and the TT genotype was a risk factor for clopidogrel resistance (*p* <0.05, OR = 1.411; Figure 1).

**TABLE 2:** Relationship between the selected single‐nucleotide polymorphisms (SNPs) of insulin signal pathway genes and clopidogrel‐resistant risk

Gene	Genotype	Non‐CR Case	CR Ctrl	OR (95% CI)	*p*‐value
PLCB1 rs67702392	CC	1	0	0.991	0.358
	TC	25	19	1.138	0.704
	TT	88	77	0.962	0.596
PLCB1 rs2743173	CC	66	44	1.263	0.081
	TC	42	47	0.753	0.077
	TT	6	5	1.011	0.986
PLCB1 rs6056209	GG	68	69	0.83	0.064
	AG	43	23	1.574	0.032
	AA	3	4	0.632	0.537
GNAS rs7121	CC	1	9	0.094	0.004
	TC	50	41	1.027	0.867
	TT	63	46	1.153	0.288
GNAS rs13831	GG	73	37	1.661	<0.001
	AG	37	50	0.623	0.004
	AA	4	9	0.374	0.079
GNAS rs114936111	GG	13	9	1.216	0.633
	AG	65	45	1.216	0.143
	AA	36	42	0.722	0.069
KCNMA1 rs4979906	GG	21	25	0.707	0.183
	AG	67	47	1.2	0.155
	AA	26	24	0.912	0.71
CCKAR rs1800857	GG	3	5	0.505	0.331
	AG	21	36	0.491	0.002
	AA	90	55	1.378	0.001
CCKAR rs2725307	CC	12	37	0.273	<0.001
	AC	74	45	1.385	0.009
	AA	28	14	1.684	0.072
CREB5 rs11765845	GG	33	47	0.591	0.003
	AG	70	34	1.734	<0.001
	AA	11	15	0.618	0.19
CREB5 rs11772815	GG	52	40	1.095	0.566
	AG	54	44	1.033	0.824
	AA	8	12	0.561	0.178
GCK,YKT6 rs4607517	GG	56	67	0.704	0.002
	AG	48	23	1.757	0.006
	AA	10	6	1.404	0.493
YKT6, GCK rs6975024	CC	10	10	0.842	0.686
	TC	59	28	1.774	0.001
	TT	45	58	0.653	0.002
PCLO rs2522833	CC	50	44	0.957	0.774
	AC	51	46	0.934	0.645
	AA	13	6	1.825	0.195
STX1A rs875342	GG	68	52	1.101	0.424
	AG	43	37	0.979	0.903
	AA	3	7	0.361	0.114
GNA11 rs11085000	GG	18	14	1.083	0.809
	TG	43	62	0.584	<0.001
	TT	53	20	2.232	<0.001
GCG rs5645	GG	58	78	0.626	<0.001
	AG	56	10	4.716	<0.001
	AA	0	8	1.091	0.002
RAPGEF4 rs17746510	GG	21	19	0.931	0.801
	TG	62	37	1.411	0.022
	TT	31	40	0.653	0.027
CREB3 rs10814274	CC	35	20	1.474	0.105
	TC	60	40	1.263	0.113
	TT	19	36	0.444	0.001

At other sites where multiple genotypes were statistically significant, a comparison between the related alleles was conducted. As shown in Table 3, *GCG rs5645* was confirmed including a relationship between genotypes containing A or G and clopidogrel resistance. No clear relationship was noted between other sites and clopidogrel resistance.

**TABLE 3:** The relationship between multiple genotype‐positive nucleotide sites and clopidogrel resistance

	CR	N‐CR	*X* ^2^	*p* value
rs13831 (GNAS)				
AA	9	4	3.088	0.079
GG + AG	87	110		
GG	73	37	13.579	<0.001
AA + AG	41	59		
A	45	68	13.03	<0.001
G	183	124		
rs2725307 (CCKAR)				
AA	14	28	3.243	0.072
AC + CC	82	86		
CC	37	12	22.865	<0.001
AA + AC	59	102		
A	73	130	15.062	<0.001
C	119	98		
rs11765845 (CREB5)				
AA	15	11	1.796	0.19
GG + AG	81	103		
GG	47	33	8.849	0.0029
AA + AG	49	81		
A	64	92	2.199	0.138
G	128	136		
rs4607517 (GCK,YKT6)				
AA	6	10	0.471	0.493
GG + AG	90	104		
GG	67	56	9.175	0.0025
AA + AG	29	58		
A	35	68	7.571	0.0059
G	157	160		
rs6975024 (YKT6,GCK)				
CC	10	10	0.164	0.686
TC + TT	86	104		
TT	58	45	9.146	0.0025
CC + TC	38	69		
T	144	149	4.6	0.032
C	48	79		
rs11085000 (GNA11)				
GG	14	18	0.0587	0.809
TG + TT	82	96		
TT	20	53	15.128	<0.001
GG + TG	76	61		
G	90	79	6.479	0.011
T	102	149		
rs5645 (GCG)				
AA	8	0	9.876	0.0017
GG + AG	88	114		
GG	78	58	21.067	<0.001
AA + AG	18	56		
A	26	56	8.056	0.0045
G	166	172		
rs17746510 (RAPGEF4)				
GG	19	21	0.064	0.801
TG + TT	77	93		
TT	40	31	4.878	0.0272
GG + TG	56	83		
T	117	124	1.829	0.176
G	75	104		

Note

The significant values are marked in bold (*p* ≤ 0.05).

## 4 DISCUSSION

A recent TRITON‐TIMI trial showed that prasugrel is superior to clopidogrel with a lower incidence of the combined endpoint of cardiovascular death.^16^, ^17^ In the PLATO trial, ticagrelor provided more potent platelet inhibition than clopidogrel for patients diagnosed with STEMI and treated with percutaneous coronary interventions (PCI).^18^ Nonetheless, despite the superior efficacy of ticagrelor and prasugrel, clopidogrel remains a major antiplatelet agent used in the treatment of patients with acute coronary syndrome (ACS) or undergoing percutaneous coronary interventions in Asia.

Clopidogrel regulates platelet activation and aggregation by irreversibly binding to the platelet P2Y12 receptor. Ellis KJ reported that the efficacy of platelet inhibition depends on clopidogrel activating metabolite by *CYP2C19*.^19^ Individuals with non‐functional copies of the *CYP2C19* gene exhibited no enzyme activity and could not convert clopidogrel through the *CYP2C19* pathway. This indicates an increased risk of major adverse cardiovascular events.^20^ Notably, Chinese have higher *CYP2C19* poor metabolizers than Caucasians and African Americans.^21^ Other genes including *ABCB1*,^22^ *P2Y12*,^23^ *PEAR1*,^24^ and *GPIIIA* ^25^ potentially regulate clopidogrel metabolism.

Previous studies have confirmed the presence of loci in the analysis of multiple genotype‐positive loci. Dysregulation of PLCB1 is a potential mechanism that links circadian rhythm disruption to pancreatic dysfunction.^26^ T C Zhou showed that PLCB1 regulates the energy or glucose homeostasis in the development of type 2 diabetes in one family.^27^ They also revealed that insulin secretion is potentially enhanced via the stimulation of particular G_q_ protein‐coupled receptors by PLCB1.^28^

Among the *GNAS rs7121* nucleotide polymorphisms, previous studies indicated that *rs7121* is linked to obesity.^12^ Several lines of evidence indicate that obesity is a risk factor for reduced clopidogrel reaction in serum. The inflammatory state associated with obesity inhibits the activity of cytochrome P450 enzymes and increases the multiple mechanisms of platelet turnover. All of the abovementioned mechanisms are potentially responsible for a decreased reactivity of clopidogrel.^29^, ^30^ As such, we speculate that the CC genotype of *GNAS rs7121* regulates clopidogrel resistance, thereby affecting the responsiveness of related drugs via inflammation related to body obesity.

Interestingly, the *rs4607517* polymorphism of the *GCK* gene is closely related to diabetes, whether in the general population or pregnant women.^31^, ^32^, ^33^ Further, many studies confirmed that patients with hyperglycemia or diabetes have an increased chance of clopidogrel resistance, that is, diabetes weakens the responsiveness to antiplatelet drugs (particularly clopidogrel). In the middle, obesity may also play an important role.^34,35^ Previous study showed that the increased methylation in GCK indicated a risk of the clopidogrel resistance in male patients with dyslipidemia.^36^ This is related to the previous results of *GNAS rs7121*, and there might be a mechanism of related influence between them, not a unilateral relationship.

On the other hand, *RAPGEF4 rs17746510* is associated with cognitive decline in Chinese patients with Alzheimer's disease. It is also significantly associated with mood disorders including anxiety.^37^ Anxiety is related to platelet function and responsiveness to drugs.^38^ Therefore, we hypothesize that the relationship between *rs17746510* and clopidogrel resistance is potentially caused by the long‐term effect on mood. However, information on precise related mechanisms is limited. The PERIOD3 (PER3) as the rhythm regulation gene was proved helpful to assess the clopidogrel resistance.^39^ Other SNPs have been confirmed to be related to clopidogrel resistance; however, their reasons and mechanisms are unclear.

Interindividual response heterogeneity is linked to several factors including age, renal and liver function, diabetes mellitus, and smoking by upregulation of platelet‐signaling pathways. Hurst M Hall et al.^40^ reported that increased platelet activation and aggregation are attributed to several metabolic illnesses including hyperglycemia, insulin resistance, and dyslipidemia in DM The phenomenon of decreased circulating active metabolites, while maintaining normal clopidogrel have been noted in patients with DM.^41^ Moreover, additional mechanisms influence clopidogrel resistance caused by the loss of sensitivity to insulin.^42^

## CONFLICT OF INTEREST

The authors declare no conflict of interest.

## Data Availability

All data and models generated or used during the study are available in a repository or online in accordance with funder data retention policies (Provide full citations that include URLs or DOIs.)
